# The common factor of conspiracy mentality: a bifactor analysis of four short-form measures of general conspiracy thinking

**DOI:** 10.3389/fpsyg.2026.1941055

**Published:** 2026-10-02

**Authors:** Steven M. Smallpage, Alexander L. Jensen, Robert L. Askew

**Affiliations:** 1 School of Politics, Security, and International Affairs, College of Sciences, University of Central Florida, Orlando, FL, United States; 2 Department of Psychology, Stetson University, DeLand, FL, United States

**Keywords:** bifactor model, conspiracist ideation, conspiracy mentality, construct validity, paranormal belief, partisanship, psychometrics, scale interchangeability

## Abstract

**Introduction:**

Conspiracy theory researchers increasingly rely on general measures of “conspiracy mentality” to explain belief in specific conspiracy theories. A growing consensus holds that the leading short-form scales are effectively interchangeable. This recommendation assumes that the scales measure the same underlying trait. We conduct a three-step test to determine whether four widely used measures are interchangeable in reflecting a single common conspiracy-mentality factor.

**Methods:**

Using a nationally representative U.S. sample, we conducted a bifactor analysis partitioning the common and unique variance of the American Conspiracy Theories Scale (ACT), the Conspiracy Mentality Questionnaire (CMQ), the General Measure of Conspiracism (GMC), and the Generic Conspiracist Beliefs Scale (GCB-5). We compared the general and scale-specific factors on reliability and on structural, convergent, discriminant, and criterion validity, including prediction of belief in individual conspiracy theories.

**Results:**

A single common factor accounted for most of the variance across all four short-form scales and evidenced comparable performance in terms of internal consistency, test–retest reliability, and in structural, convergent, discriminant, and criterion validity. The GCB-5 retained meaningful but apparently additional paranormal content even after controlling for the common factor of conspiracy mentality. The GMC’s unique residual correlated with partisan-coded beliefs about vaccines, SARS (COVID-19), climate change, and the Obama “birther” conspiracy. The GMC also tended to produce less extreme values than the other three scales at both ends of the trait, diverging from the common factor by over a standard deviation.

**Discussion:**

The four short-form generic scales are not strictly interchangeable. While these scales share a common conspiracy mentality factor, two of them carry measurable, orthogonal content that inflates their associations with beliefs in specific conspiracy theories: paranormal content in the GCB-5, and partisan content in the GMC. The GMC also maps observed scores onto the common factor differently from the other three scales, particularly at the extremes of the trait. When measuring conspiracy thinking, researchers should treat the choice of scale as a deliberate decision and should not assume estimates obtained with different scales are equivalent. Further, we recommend that researchers consider controlling for paranormal thinking or partisanship when using the GCB-5 or the GMC, respectively.

## Introduction

Conspiracy theory researchers have increasingly adopted generic measures of a putative latent “conspiracy mentality,” defined as the “very basic worldview that the fate of the world is determined by plans hatched in secret” (Imhoff et al., 2022, p. 4). These measures rely on general items, such as “things do not happen by accident,” that are intended to measure the underlying disposition that leads people to endorse specific conspiracy theories. The appeal of this approach is that it avoids the problem of specific content: the endorsement of any specific conspiracy theory reflects both a latent conspiracy mentality worldview and content specific to that theory, which may vary in meaning and salience depending on time, place, political context, and respondent familiarity (Brotherton et al., 2013; Bruder et al., 2013; Uscinski and Parent, 2014; Imhoff et al., 2022; Smallpage et al., 2023; Imhoff, 2024). For example, a specific conspiracy theory item about a stolen election likely captures both the respondent’s latent disposition to believe a conspiracy theory and the respondent’s partisanship (Smallpage et al., 2017; Enders et al., 2020). In this way, using items that are specific conspiracy theories to measure an underlying predisposition toward conspiracism may be a “content-contaminated” indicator of the underlying conspiracy mentality, since endorsement may be a function of other factors too (such as partisanship, in our example) (Imhoff et al., 2022, p. 3). Generic items are meant to strip this contamination away by not referencing a particular theory, reflecting only the abstract worldview that drives conspiracy mentality.

From this perspective, if conspiracy mentality is a trait that manifests in dispositional individual differences, then it should be stable, continuously distributed, and *reflected* in its indicators rather than *constituted* by them (Imhoff et al., 2022). Imhoff (2024) argues for a “reflective model whereby the endorsement of each specific conspiracy belief merely reflects the latent variable,” analogous to the way personality traits are reflected in their indicators (Imhoff, 2024, p. 60). Further, as Imhoff (2024) notes, if “conspiracy mentality is just a summative characterization of the way participants respond to specific items, any different scale (or subset of items) will form different *conspiracy mentalities*” (Imhoff, 2024, p. 60) and not a single conspiracy mentality trait. Instead, the prevailing assumption of the field is that the generic scales are reflective of the underlying conspiracy mentality trait, such that while there may be multiple measures each should recover the same underlying trait, and whatever is unique to each scale should be mere noise (Imhoff, 2024, p. 60).

A number of general conspiracy mentality scales are now available to researchers to capture this underlying disposition to believe conspiracy theories, such as the Generic Conspiracy Beliefs Scale (GCB-15; Brotherton et al., 2013; GCB-5, Kay and Slovic, 2023), American Conspiracy Theories (ACT) Scale (Uscinski and Parent, 2014), General Measure of Conspiracism (GMC; Drinkwater et al., 2012), and the Conspiracy Mentality Questionnaire (CMQ; Bruder et al., 2013). Yet, the use of any one of these scales to measure a common underlying predisposition rests on the fundamental assumption of classical test theory – that the variance in scale items is a composite of two things: “true” score variance attributable to the target trait and random measurement error. Residual systematic variance beyond these two sources may be an indicator of contamination which could not only undermine a measurement exercise but may systematically differentially report effects in studies employing some measures. This same fundamental assumption supports the reflective model of conspiracy mentality.

This reflective assumption is well-supported at the level of individual scales, as recent empirical work demonstrates that some short-form generic conspiracy mentality measures predict endorsement of specific conspiracy theories (Imhoff et al., 2022; Imhoff, 2024), and that each of the predominant generic scales is unidimensional, stable, and durable over time (Kay and Slovic, 2026). Comparing the GCB-5, ACT, CMQ, GMC, and One-Item Measure of Conspiracy (1CM), Kay and Slovic (2026, 297) conclude that they are all “reliable and valid measures of conspiracist ideation.” On this basis, they recommend that “researchers use whichever of the five measures they prefer” (Kay and Slovic, 2026, 297). These studies establish that the generic scales, individually, have sound criterion validity (i.e., they are strongly correlated with beliefs in specific conspiracy theories) and have reliability in line with expectations for a reflective model. Importantly, what these studies do *not* establish – and no other empirical studies to our knowledge have either – is whether the popular generic conspiracy mentality scales measure the *same* latent trait. In other words, the fact that each scale independently predicts belief in specific conspiracy theories is consistent with the reflective model’s assumption that they share a common trait, but it is also equally consistent with the possibility that each generic scale reflects different aspects of conspiracy mentality, such that there may be distinct dispositions—multiple “conspiracy mentalities,” to borrow Imhoff’s (2024) phrase—that converge on the same outcome (belief in specific conspiracy theories).

While each of the four short-form generic scales were designed to measure the same latent conspiracy mentality trait, the scales and their constituent items differ substantially from one in content and intent (Kay and Slovic, 2026). The authors of short-form generic scales used different approaches to item-generation (see Table 1 for a full list of these items). Following the reflective model, the CMQ takes an abstract dispositional approach, deliberately stripping items of concrete content so that the scale indexes a generalized attitude toward secret group action rather than any substantive belief about specific secret groups (Bruder et al., 2013). Similarly, the ACT includes no specific references to actors or events, locating the disposition in the broad domain of power and politics (Uscinski and Parent, 2014). The GMC, by contrast, operates at a different level of abstraction altogether, asking respondents about their recognition and endorsement of the category of things called “conspiracy theories,” rather than asking about conspiratorial activity itself (Drinkwater et al., 2012). The GMC’s items therefore index an orientation toward the label “conspiracy theory” as much as toward the phenomenon it names, a feature Kay and Slovic (2026) themselves note, but set aside, as potentially problematic because what counts as a “conspiracy theory” varies across contexts. Rather than abstracting away from conspiracy content altogether, the 15-item GCB samples across the five thematic domains to measure the latent conspiracy ideation factor: government malfeasance, malevolent global conspiracies, extraterrestrial cover-ups, threats to personal wellbeing, and control of information (Brotherton et al., 2013). The authors of the original 15-item GCB designed these thematic areas as facets of conspiracy mentality, ensuring content coverage, but recommended that the scale be modeled as unidimensional since they intercorrelate in the real world (Brotherton et al., 2013, p. 7). They also explicitly acknowledged the tension in the GCB instrument itself: between a bottom-up thematic approach across five different areas, yet intended to measure a single, abstract conspiracy mentality trait.

**Table 1:** Item content from the ACT, CMQ, GCB-5, and GMC.

Item ID	Item wording
ACT.1	Even though we live in a democracy, a few people will always run things anyway.
ACT.2	The people who really “run” the country are not known to the voters.
ACT.3	Big events like wars, the recent recession, and the outcomes of elections are controlled by small groups of people who are working in secret against the rest of us.
ACT.4	Much of our lives are being controlled by plots hatched in secret places.
CMQ.1	Many very important things happen in the world, which the public is never informed about.
CMQ.2	Politicians usually do not tell us the true motives for their decisions.
CMQ.3	Government agencies closely monitor all citizens.
CMQ.4	Events which superficially seem to lack a connection are often the result of secret activities.
CMQ.5	There are secret organizations that greatly influence political decisions.
GCB.1	The government permits or perpetrates acts of terrorism on its own soil, disguising its involvement.
GCB.2	Evidence of alien contact is being concealed from the public.
GCB.3	New and advanced technology which would harm current industry is being suppressed.
GCB.4	Certain significant events have been the result of the activity of a small group who secretly manipulate world events.
GCB.5	Experiments involving new drugs or technologies are routinely carried out on the public without their knowledge or consent.
GMC.1	Conspiracy theories accurately depict real life events.
GMC.2	The information contained within conspiracy theories is generally true.
GMC.3.r	When I hear conspiracy theories, I feel they are untrue.
GMC.4.r	Conspiracy theories contain information, which has proved to be false.
GMC.5	I have heard several conspiracy theories, which I believe to be true.

Whether a given item in a scale counts as a strength or a defect depends on what the scale is meant to reflect. While the CMQ, ACT, GMC, and GCB-15 differ in item content, they are intended to measure the latent “the general tendency” (Brotherton et al., 2013, p. 1), the “the general propensity” (Bruder et al., 2013, p. 2), the “general belief” (Drinkwater et al., 2012, p. 61), and the “general concepts” (Uscinski and Parent, 2014, p. 79) that underwrite beliefs in specific conspiracy theories. So, when judging whether a scale is interchangeable, we must investigate how well it measures that latent trait and nothing else. But whether unique content is a defect cannot be determined *a priori*, since, for example, the breadth of items as found in the GCB-15 or the GCB-5 could be a virtue. Indeed, when designing the GCB-15, Brotherton et al. (2013) report that different facets predict different specific conspiracy theories, presenting this as evidence of criterion validity.

Recently, Kay and Slovic (2026) also found that the GCB-5 had the largest association with the Belief in Conspiracy Theories Inventory (BCTI) relative to the other measures, and noted that this correlation may be inflated by uniquely similar content in items on the GCB-5 and the BCTI (e.g., “Evidence of alien contact is being concealed from the public” and “In July 1947, the US military recovered the wreckage of an alien craft from Roswell, NM and covered up the fact”, respectively; full question wordings for these scales can be found in Tables 1, 2). Kay and Slovic (2026) argue that while the GCB-5 may have “rogue variance”, that may in fact be a unique virtue of the scale since it was “designed to capture the 5 themes of conspiracist ideation” and thus may be “better able to capture the full breadth of the conspiracist ideation construct,” relative to the other generic conspiracy mentality scales.

**Table 2:** Item content from the BCTI.

Item ID	Item wording
BCTI.1	A powerful and secretive group, known as the New World Order, are planning to eventually rule the world through an autonomous world government, which would replace sovereign government.
BCTI.2	SARS (Severe Acute Respiratory Syndrome) was produced under laboratory conditions as a biological weapon.
BCTI.3	The US government had foreknowledge about the Japanese attack on Pearl Harbour but allowed the attack to take place so as to be able to enter the Second World War.
BCTI.4	US agencies intentionally created the AIDS epidemic and administered it to Black and gay men in the 1970s.
BCTI.5	The assassination of Martin Luther King, Jr., was the result of an organized conspiracy by US government agencies such as the CIA and FBI.
BCTI.6	The Apollo moon landings never happened and were staged in a Hollywood film studio.
BCTI.7	Area 51 in Nevada, US, is a secretive military base that contains hidden alien spacecraft and/or alien bodies.
BCTI.8	The US government allowed the 9/11 attacks to take place so that it would have an excuse to achieve foreign (e.g., wars in Afghanistan and Iraq) and domestic (e.g., attacks on civil liberties) goals that had been determined prior to the attacks.
BCTI.9	The assassination of John F. Kennedy was not committed by the lone gunman, Lee Harvey Oswald, but was rather a detailed, organized conspiracy to kill the President.
BCTI.10	In July 1947, the US military recovered the wreckage of an alien craft from Roswell, New Mexico, and covered up the fact.
BCTI.11	Princess Diana’s death was not an accident, but rather an organized assassination by members of the British royal family who disliked her.
BCTI.12	The Oklahoma City bombers, Timothy McVeigh and Terry Nichols, did not act alone, but rather received assistance from neo -Nazi groups.
BCTI.13	The Coca Cola company intentionally changed to an inferior formula with the intent of driving up demand for their classic product, later reintroducing it for their financial gain.
BCTI.14	Special interest groups are suppressing, or have suppressed in the past, technologies that could provide energy at reduced cost or reduced pollution output.
BCTI.15	Government agencies in the UK are involved in the distribution of illegal drugs to ethnic minorities.
BCTI.16	Some airplanes release chemical/biological agents intended to control the human population.
BCTI.17	Former President Barack Obama was born in Kenya, not Hawaii.
BCTI.18	Many victims of mass shootings are, in reality, actors.
BCTI.19	Since 1998, scientists have been trying to cover up the fact that certain vaccines cause autism.
BCTI.20	Global warming is an invented concept, created to suppress economic growth.
BCTI.21	Despite what NASA says, the earth is neither a sphere nor a globe; it is a flat plane.

There are two ways to theorize whether the GCB-15’s and the GCB-5’s unique variance is a feature or a defect of the scale. On the one hand, the unique variance is seen as a feature, such that some scholars have suggested that the notion of a single underlying conspiracy mentality trait should be revised in favor of a multidimensional approach (Nera, 2024a,b; Denovan et al., 2026). Recent research into the psychometric properties of the GCB-15, for example, has suggested that the scale should be understood as multidimensional, though with highly correlated subfactors (Denovan et al., 2026). On the other hand, it is seen as a defect, since the GCB-15’s predictive power may reflect the same kind of “content-contamination” in specific conspiracy belief scales identified by Imhoff et al. (2022, p. 3): the endorsement of any specific conspiracy theory belief reflects both a general tendency to believe in conspiracy theories and also “specific context with a specific target,” and therefore is not “just an expression of the tendency to believe in conspiracies per se” and “might just be a conspiracy-unrelated sentiment phrased in the form of a conspiracy theory” (Imhoff et al., 2022, 3). In this case, since the BCTI contains conspiracy beliefs about extraterrestrials, and the GCB-5 includes an extraterrestrial cover-up item, then the GCB-5 should be more correlated with the BCTI because it is also capturing a respondent’s conspiracy mentality and the respondent’s disposition toward paranormal beliefs (Nera, 2024a,b; Denovan et al., 2026).

Regardless, the only way to begin to adjudicate whether generic scales of conspiracy mentality, and the GCB-5, are better or worse reflections of the underlying trait—and only the underlying trait—is to disentangle what is shared and what is unique to each of the conspiracy mentality scales. Thus, the central empirical question of this paper is whether the conspiracy mentality scales are interchangeable with respect to reflecting a single common latent trait, as the prevailing reflective model of conspiracy mentality assumes. To answer this question, we operationalize interchangeability as the combination of three conditions. Following Imhoff et al. (2022), the reflective model’s basic requirement is that the scales share a single latent trait. If each scale is an indicator of a single underlying conspiracy mentality, then the four scales modeled together should converge into a common factor that accounts for the bulk of their covariation. We therefore first confirm that each scale is individually unidimensional, then model the four scales jointly into a bifactor structure where we can derive a general factor from the covariance among all items across all scales, alongside orthogonal specific factors capturing the residual variance that remains unique to each scale. Our first hypothesis (H1) holds that the scales reflect a single common conspiracy mentality factor that dominates their scale-specific variance and is itself a valid and reliable measure of the trait that is associated with established correlates of conspiracy thinking. Confirming H1 is the first step toward establishing interchangeability among the scales.

*H1*: The four scales reflect a single common conspiracy mentality factor that dominates their scale-specific variance and is itself a valid and reliable measure of the trait.

Having demonstrated a shared, common factor explains the correlations among the four scales, our second hypothesis (H2) predicts that, given the shared factor, the scale-specific residuals should not predict specific conspiracy beliefs beyond the shared factor (Imhoff, 2024). This is an important step toward establishing interchangeability because, when it comes to predicting specific conspiracy beliefs, it requires that the scales be reflections of only the common factor and random measurement error. If, however, a scale-specific residual factor predicts specific conspiracy belief endorsement beyond the common factor, then that demonstrates that the scale is reflecting something in addition to the shared trait. In other words, if a generic scale’s residuals predict specific conspiracy beliefs, this would not be in line with the reflective model assumption, which would mean the scales are not strictly interchangeable with each other.

*H2*: The residual variance in each scale-specific factor does not systematically predict individual conspiracy beliefs beyond the common factor.

Finally, we also have a theoretical expectation for the relationship between the shared variance of each scale and the common factor. Our third hypothesis (H3) addresses the practical concern that a scale cannot be considered interchangeable if it differentially reports a respondent’s true level of the common trait in relation to the other scales. This holds true even if the scale successfully reflects the shared trait (H1) and contains no unique residual variance that explains conspiracy belief endorsement (H2). In other words, the scale of the common factor and the observed scores of each individual scale should share a monotonic linear relationship, placing individual trait scores consistently across both metrics. Therefore, H3 predicts that each scale’s scores have a monotonic linear relationship with the general factor. This is a necessary final step to establish interchangeability because scales could differ in how strongly they reflect the common trait of conspiracy mentality.

*H3*: Each scale’s scores should be monotonically related to the common factor and should order respondents similarly across its range.

## Materials and methods

### Participants and procedures

The present analyses use secondary data from Kay and Slovic’s (2026) study of short-form conspiracist mentality measures. The original data and scripts are publicly available via the authors’ OSF page.^1^ The original data collection was reviewed and approved by the Human Subjects Review Committee at Union College (E23033) and all participants provided informed consent (Kay and Slovic, 2026). Because their original article describes the sample and procedures in detail, we briefly summarize only the features most relevant to our reanalysis. Kay and Slovic recruited a nationally representative sample of U.S. participants through the online survey company Prolific. Prior methodological work has found that Prolific generally produces high-quality responses relative to several other online platforms and panels (Peer et al., 2017, 2022; Douglas et al., 2023).

Participants completed an initial online survey containing conspiracy mentality measures and demographic items. After the correction for missing and distracted respondents, as described by Kay and Slovic (2026), the initial sample included 492 participants; of whom, 389 completed a follow-up survey approximately 2 weeks later. This two-wave design allowed Kay and Slovic (2026) to evaluate the internal consistency, short-term test–retest reliability, and construct validity of five short-form conspiracist ideation measures. The sample was quota-balanced on key demographic and political characteristics. Kay and Slovic (2026) report that 48.98% of participants identified as women and 49.80% identified as men; the sample was also balanced by partisanship, with 46.75% identifying as Democrats and 46.54% identifying as Republicans.

#### Description of measures

We examine the construct validity of four short form conspiracy mentality scales by correlating them with 21 specific conspiracies and other related concepts. In this section, we briefly examine each of the constructs – though, for a detailed overview of these measures, including the background on why and how each was developed, see Kay and Slovic (2026). All item wordings for the generic scales of conspiracy mentality used in this study can be found in Table 1; the item wordings for the specific conspiracy theory beliefs used in this study can be found in Table 2.

#### Conspiracy mentality measures

One of the virtues of the generic attempts to measure the conspiracy mentality instead of specific conspiracy theories is that the former are more abstract, context-free questions that “taps into meaningful differences in the very basic worldview that the fate of the world is determined by plans hatched in secret,” and the latter may be “content-contaminated” with other context-specific factors (Imhoff et al., 2022, p. 3). The general measures are supposed to contain items that are, therefore, more abstract. The American Conspiracy Thinking (ACT) Scale (Uscinski and Parent, 2014) is comprised of four general items that capture belief in covert control of national events by a secret group of elites. Three of the four items of the ACT explicitly capture an element of political/governmental secrecy (ACT items 1–3, Table 1), and the fourth item captures a broader belief (without reference to politics/government) that “much of our lives” are influenced by the private plotting of others (Uscinski and Parent, 2014). The Conspiracy Mentality Questionnaire (CMQ; Bruder et al., 2013) assesses a general tendency to attribute major societal events to the coordinated, secretive scheming of elites with a 5-item index. Of the five items, three connect explicitly to political beliefs: one gauges belief that politicians have secret motives, the next gauges belief in covert government monitoring of citizens, and the third gauges belief that “secret organizations” shape political outcomes (Bruder et al., 2013). The other two items of the CMQ assess broad beliefs that citizens are kept unaware of “many important things [that] happen in the world” and that seemingly otherwise unrelated/accidental features of the world are the product of coordination. The CMQ is the most widely used short form measure in the quantitative literature on conspiracy beliefs (Kay and Slovic, 2026) which has produced an extensive and varied body of validity evidence. The Generic Conspiracist Beliefs Scale (GCB) captures a disposition toward conspiracist thinking across five domains. Originally constructed as a 15-item scale from 75 original items (GCB-15; Brotherton et al., 2013), the GCB includes general measures that capture belief in “government malfeasance” conspiracies, “malevolent global” conspiracies, belief in “extraterrestrial cover-ups,” “personal wellbeing” (PW) conspiracies, and “control of information” (CI) conspiracies. Recent work has also validated the condensed 5-item version of the GCB (GCB-5; Kay and Slovic, 2023; Dagnall et al., 2023). Another 5-item index, the “General Measure of Conspiracism” (GMC) by Kay and Slovic (2026), was originally developed in a study of the association between conspiracy and paranormal beliefs (Drinkwater et al., 2012). The GMC differs substantively from the other conspiracy mentality measures. Rather than probing general attitudes, the items contained in this index simply ask respondents how credible they find “conspiracy theories” discursively, making responses dependent on personal interpretations of what constitutes a conspiracy. Four of the five items gauge awareness of undefined “conspiracy theories” and explicitly assess judgement of their truthfulness as a category of belief. The fifth item in the index assesses the “accuracy” of conspiracy theories. (Throughout the Proposed Analysis and Results sections, we will refer to the GCB-5 as simply “GCB.”)

#### Belief in conspiracies theories inventory

The Belief in Conspiracy Theories Inventory (BCTI) (Swami et al., 2010, 2011; Kay and Slovic, 2023) measures belief in *specific* conspiracy theories, as opposed to an underlying conspiracy mentality. Designed to capture belief in specific conspiracies that would “be recognized by an international audience” (Swami et al., 2011), the BCTI was created initially by Swami et al. (2010) study examining the precursors of 9/11 conspiracy beliefs. It was subsequently refined by the Swami et al (2011) as the BCTI-15 used by Kay and Slovic (2023, 2026). Kay and Slovic (2023) then expanded the BCTI-15 to the 21-item version in their 2023 paper, adding six new items (Items 16–21 in Table 2) that capture belief in chem trails, in the Obama “birther” conspiracy, that mass shooting victims are “paid actors,” that vaccines cause autism, that “global warming” was invented to suppress economic growth, and belief in the “flat earth” conspiracy. The BCTI uses a nine-point answer set gauging respondents’ belief in their authenticity (“completely false” to “completely true”).

#### Correlates of conspiracy mentality

The Schizotypal Personality Questionnaire’s (SPQ) “Odd Beliefs or Magical Thinking” Subscale (Raine, 1991) is a 7-item subscale capturing the propensity to hold supernatural or magical beliefs, with sample content touching on phenomena such as ESP, clairvoyance, and psychic forces. Its inclusion tests whether endorsement of conspiracy theories aligns with a more general openness to paranormal phenomena or otherwise unconventional explanations of reality. The original SPQ index was constructed from binary measures where respondents were asked yes/no questions regarding their beliefs in supernatural or magical beliefs.

The Persecution and Deservedness Scale (PDS) (Melo et al., 2009) is a 10-item scale that captures agreement with statements of paranoid ideation - asking respondents about fears that others may be working against them. It serves as a check on whether conspiracist thinking tracks with a broader tendency toward suspicion of others’ intentions.

The Agnew’s Anomie Scale (AGN) (Agnew, 1980) is an 8-item scale capturing *anomie* - a sense that social bonds and trust have eroded. This scale asks questions which gauge a sense of no longer knowing whom one can rely on and feeling a lack of control over one’s situation in life. The scale’s items probe whether conspiracist belief is bound up with feelings of social disconnection. Agnew (1980) does not clearly identify the scale of the response options for the original measure, though the scale from which it was adapted (Srole, 1956) used employed a simple three-level (agreement/disagreement/unsure) response set.

The Need for Chaos (NFC) Scale (Arceneaux et al., 2021) is a 7-item scale with anti-social content (e.g., wanting society to “burn”). Arceneaux et al. (2021) assert that this need for chaos is a bid of attention through an act of subversion (2021). While one of the items explicitly taps the “need” for chaos, the other items gauge endorsement of destruction and related anti-establishment attitudes. The original NFC employed a 7-point Likert-style (dis)agreement response set.

The Denialism (DEN) Scale (Uscinski et al., 2020) was developed for a study examining the drivers of COVID-19-related conspiracy beliefs. The developers of this 4-item scale assert that it measures the extent to which individuals “reject expert, authoritative information” (Uscinski et al., 2020). The items on the denialism scale assess the extent to which respondents believe there is a hidden truth behind mainstream narratives, while only one of the items explicitly mentions government (representing authoritative expertise). The items of the Denialism scale were originally gauged through a 5-point Likert-style scale of (dis)agreement.

Finally, the Illusory Pattern Perception (IPP) Scale (Dagnall et al., 2007; van Prooijen et al., 2018) is a measure of people’s tendency to detect order or patterns in genuinely random information. Unlike the other attitudinal scales examined here, the IPP focuses more on judgement. Respondents judged how random or determined each of 10 individual series of 10 (randomly generated) coin-flip sequences appear, along with a final evaluation of all 100 coin-flips in a single composite sequence. The arithmetic mean of these observations formed respondents’ individual IPP score. This scale served as a test of whether conspiracist belief stems partly from over-detecting meaningful patterns in noise.

Kay and Slovic (2026) administer these measures of conspiracy mentality and their correlates with a common seven-point, fully labeled Likert response set (“strongly agree” to “strongly disagree”). Two measures depart from this format: the IPP Scale uses a seven-point scale with only the poles labeled (“completely random” and “completely determined”), and BCTI uses a nine-point scale with only the poles labeled “completely false” and “completely true.” For more information on the development of these scales and the psychometric evidence supporting their use, see the extended discussion in Kay and Slovic’s (2026) paper. The item wordings for these scales are listed in Table 3.

**Table 3:** Item content from the likely correlates of conspiracy mentality.

Item ID	Item wording
PDS.1	There are times when I worry that others might be plotting against me.
PDS.2	I often find it hard to think of anything other than the negative ideas others have about me.
PDS.3	My friends/others often tell me to relax and stop worrying about being deceived or harmed.
PDS.4	Every time I meet someone for the first time, I’m afraid they’ve already heard bad things about me.
PDS.5	I’m often suspicious of other people’s intentions towards me
PDS.6	Sometimes, I just know that people are talking critically about me.
PDS.7	There are people who think of me as a bad person.
PDS.8	People will almost certainly lie to me.
PDS.9	I believe that some people want to hurt me deliberately.
PDS.10	You should only trust yourself.
SPQ.1	I’ve had experiences with the supernatural.
SPQ.2	I believe in telepathy (mind-reading).
SPQ.3	I am sometimes sure that other people can tell what I am thinking.
SPQ.4	I believe in clairvoyancy (psychic forces, fortune telling).
SPQ.5	Other people can feel my feelings when they are not with me.
SPQ.6	I’ve had experiences with astrology, seeing the future, UFOs, ESP, or a sixth sense.
SPQ.7	I have felt that I was communicating with another person telepathically (by mind-reading).
AGN.1	I don’t blame anyone for trying to grab all they can get in this world.
AGN.2	It’s no use worrying my head about public affairs; I can’t do anything about them anyway.
AGN.3	Most people make friends because friends are likely to be useful to them.
AGN.4	I have had more than my fair share of worries.
AGN.5	These days a person really doesn’t know who they can trust.
AGN.6	Success is more dependent on luck than on real ability.
AGN.7	There’s little use writing to public officials because they aren’t really interested in the problems of the average person.
AGN.8	There are so many ideas about what is right and wrong these days that it is hard to figure out how to live your life.
NFC.1	I get a kick when natural disasters strike in foreign countries
NFC.2	I fantasize about a natural disaster wiping out most of humanity such that a small group of people can start all over.
NFC.3	I think society should be burned to the ground.
NFC.4	When I think about our political and social institutions, I cannot help thinking ‘just let them all burn’.
NFC.5	We cannot fix the problems in our social institutions, we need to tear them down and start over.
NFC.6	I need chaos around me —it is too boring if nothing is going on.
NFC.7	Sometimes I just feel like destroying beautiful things.
DEN.1	Much of the information we receive is wrong.
DEN.2	I often disagree with conventional views about the world.
DEN.3	Official government accounts of events cannot be trusted.
DEN.4	Major events are not always what they seem.
IPP.1	THHTTHHHHH
IPP.2	TTTTTHHTTT
IPP.3	HHHHHHTHHT
IPP.4	HTTHTTHTTT
IPP.5	TTTHTTHHHT
IPP.6	HTHHTTHTHH
IPP.7	HHTTHTHTHH
IPP.8	HTHHHTTHHT
IPP.9	HHTTTHTHHH
IPP.10	THTTHTTTTT
IPP.11	Now, imagine that the above items represent 100 consecutive throws with the same coin. Again, rate how random or determined you believe the outcomes are.

### Proposed analysis

*H1*: The four scales reflect a single common conspiracy mentality factor that dominates their scale-specific variance and is itself a valid and reliable measure of the trait.

To test this hypothesis, we first fit four unidimensional factor models and inspected factor loadings and model fit indices. We used polychoric correlation matrices, given the ordinal nature of response options and a means and variance adjusted diagonally weighted least squares estimator (WLSMV) because it is a more robust estimator when factor analyzing ordinal response structures (Flora and Curran, 2004). To assess model fit, we used traditional indices (i.e., *X*^2^, CFI, TLI, RMSEA, and SRMR), but prioritized SRMR, because CFI and TLI may exhibit upward bias when modeling ordinal data with WLSMV (Xia and Yang, 2019), and SRMR has demonstrated superior performance relative to RMSEA in terms of statistical power and control of Type I error (Shi et al., 2020), particularly in models with large numbers of indicators (Maydeu-Olivares et al., 2018). We then fit a single correlated factors model to evaluate stability of model estimates and inter-factor correlations.

We then fit a bifactor model with all items loading simultaneously on a general factor and on a scale specific factor, and as per convention, we specified the general and all scale specific factors as orthogonal to each other. We then compared model fit indices from the bi-factor model to the four unidimensional models and compared the loadings on the general factor to loadings on the specific factors as assessments of structural validity. We then extracted factor scores using the Empirical Bayes Modal (EBM) approach and compared scores for the general factor to scores for the original scales in terms of convergent-, discriminant- and criterion-validity.

With respect to convergent and discriminant validity, we estimated Pearson’s *r* correlations between scale scores and scores from theoretically related measures (i.e., the SPQ, PDS, AGN, NFC, DEN, and IPP). As a separate test of criterion validity, we also compared effect size measures ( $\eta_{\text{partial}}^{2}$ and $\eta_{\text{semi}-\text{partial}}^{2}$ ) of scores from each scale when predicting magnitude of beliefs in specific conspiracy theories (mean BCTI score) in a multivariable linear regression using a type III sum of squares decomposition (Cohen and Cohen, 2003). Because multicollinearity among the predictors is likely, we chose $\eta_{\text{semi}-\text{partial}}^{2}$ as it represents the proportion of total variance in the outcome (in this case, mean BCTI score) that is *uniquely* explained by each individual predictor (in this case, variance attributable only to GCB, CMQ, GMC, ACT, or a general factor) as follows.

$$\eta_{\text{semi}-\text{partial}}^{2}=SS_{\text{unique effect}}/SS_{\text{total}}$$

We also calculated $\eta_{\text{partial}}^{2}$ which also conditions on all the other predictors, by only including the variability attributable to that unique predictor and the error variance in the denominator, as follows.

$$\eta_{\text{partial}}^{2}=SS_{\text{unique effect}}/(SS_{\text{unique effect}}+SS_{\text{error}})$$

With respect to internal consistency reliability, we estimated three complementary indices: Cronbach’s α , McDonald’s $\omega_{\text{total}}$ , and McDonald’s $\omega_{\text{hierarchical}}$ (Kalkbrenner, 2024). Cronbach’s α is one of the most popular measures of internal consistency that can be interpreted as the proportion of total score variance attributable to a common source (i.e., latent construct). One important limitation of Cronbach’s α is that it assumes that all items contribute equally to that construct (i.e., the tau equivalence assumption). In contrast, McDonald’s $\omega_{\text{total}}$ offers a more flexible, factor model-based estimate of total score reliability that relaxes this assumption by allowing items to have different factor loadings. McDonald’s $\omega_{\text{hierarchical}}$ employs a bifactor model to further isolate the proportion of variance attributable to a common general factor that partitions out variance associated with subscale-specific factors, thereby informing the degree to which a unidimensional interpretation of a scale is justified. For test–retest reliability, we estimated both Pearson’s *r* and Intraclass Correlation (ICC) version 2,1 (Shrout and Fleiss, 1979). Within a two-way random effects, absolute agreement, single-measures framework, ICC (2,1) represents the ratio of between-subject (bs) variance to the total variance, where the total variance includes both within-subject (ws) systematic variance and random error (re), as follows.

$$\mathrm{ICC}{\left(2,1\right)}_{\text{absolute}}=\sigma_{\mathrm{bs}}^{2}/(\sigma_{\mathrm{bs}}^{2}+\sigma_{\mathrm{ws}}^{2}+\sigma_{\mathrm{re}}^{2})$$

Because the items from all scales load on the general factor of the bi-factor model, it is considerably longer (i.e., 19 items) than any of the original 4- and 5-items scales. As Spearman (1910) and Brown (1910) have shown, any increased reliability of the general factor could merely be a function of the increased test length, so we included Spearman-Brown (SB) adjusted estimates for the shorter scales to enhance comparability. The only metric we did not include SB-adjustments for was $\omega_{h}$ , because $\omega_{h}$ isolates a single orthogonal dimension from a multidimensional structure and controls for every other dimension, which would undermine interpretability of subsequent adjustments based on test length.

*H2*: The residual variance in each scale-specific factor does not systematically predict individual conspiracy beliefs beyond the common factor.

In addition to the structural and criterion validity evidence related to the scale-specific factors in H1, we also the inspected the convergent, discriminant, and criterion validity evidence of scores from the scale-specific factors, because if the residual uniqueness in each scale is unrelated to the common conspiracy mentality trait, then all estimates should be at or near zero except those serving as discriminant validity. We also regressed the individual items of the BCTI onto the general and scale-specific subfactors to evaluate and identify the nature of any qualitative distinctiveness of any scale-specific subfactors over and above the general factor when predicting actual conspiracy beliefs.

*H3*: Each scale’s scores should be monotonically related to the common factor and should order respondents similarly across its range.

We then evaluated if there are any differences in a specific scale’s reflection of the common trait by plotting the factor scores from the general factor against observed factor scores from the original scales using LOESS (Locally Estimated Scatterplot Smoothing) curves.

All analyses were carried out using R version 4.5.2 [2025-10-31; (R Core Team, 2025), R-Studio version 2026.01.0+392 (Posit Team, 2026), and the ‘tidyverse’ (Wickham et al., 2019), ‘tidyr’ (Wickham et al., 2025), ‘ggplot2’ (Wickham, 2016),'ggExtra’ (Attali and Baker, 2025), ‘CTT’ (Willse, 2026), ‘psych’ (Revelle, 2025), ‘lavaan’ (Rosseel, 2012), ‘polycor’ (Fox, 2025), ‘semPlot’ (Epskamp, 2022), ‘EFAtools’ (Steiner and Grieder, 2020), ‘car’ (Fox et al., 2026), ‘effectsize’ (Ben-Shachar et al., 2020), and ‘semTools’ (Jorgensen et al., 2025) packages].

## Results

*H1*: The four scales reflect a single common conspiracy mentality factor that dominates their scale-specific variance and is itself a valid and reliable measure of the trait.

As indicated in Table 4, almost all fit indices (save RMSEA) from the four unidimensional factor models were indicative of good model fit. Factor loadings were generally good to exceptional (mean λ: 0.77, range: 0.57–0.93), with the highest average factor loadings observed for the GMC, followed by the ACT, and the CMQ, and lowest average factor loadings for the GCB (see Figure 1). When attempting to fit a preliminary correlated factors model, extreme multicollinearity was indicated by a non–positive definite Φ matrix and 3 problematic correlations among 4 latent variables ranging from 1.0 to 1.01. We considered this indicator of model misspecification as preliminary evidence that a common factor is warranted.

**Table 4:** Factor loadings, model fit indices, and reliability estimates from four unidimensional factor models and one bifactor model.

	Unidimensional factor models				Bifactor model				
	ACT	CMQ	GCB	GMC	General	s.ACT	s.CMQ	s.GCB	s.GMC
Item factor loadings									
ACT.1	0.67				0.67	0.56			
ACT.2	0.82				0.81	0.11			
ACT.3	0.89				0.90	−0.05			
ACT.4	0.83				0.84	−0.10			
CMQ.1		0.78			0.72		0.28		
CMQ.2		0.62			0.57		0.66		
CMQ.3		0.69			0.68		0.05		
CMQ.4		0.78			0.83		−0.16		
CMQ.5		0.87			0.88		0.02		
GCB.1			0.75		0.76			0.09	
GCB.2			0.57		0.54			0.41	
GCB.3			0.62		0.59			0.28	
GCB.4			0.83		0.92			−0.22	
GCB.5			0.84		0.80			0.10	
GMC.1				0.93	0.75				0.55
GMC.2				0.90	0.75				0.50
GMC.3.r				0.80	0.62				0.51
GMC.4.r				0.65	0.46				0.47
GMC.5				0.87	0.75				0.44
Fit statistics									
*X* ^2^	8.492	32.774	11.989	28.080	313.561				
*df*	2	5	5	5	133				
*p*	0.014	< 0.001	0.035	< 0.001	< 0.001				
Scaled *X*^2^	25.932	81.869	28.822	100.083	634.924				
*df*	2	5	5	5	133				
*p*	<0.001	<0.001	<0.001	<0.001	<0.001				
CFI	1.00	0.99	1.00	1.00	1.00				
Scaled CFI	0.99	0.97	0.99	0.99	0.98				
Robust CFI	0.98	0.95	0.98	0.97	0.94				
TLI	1.00	0.99	1.00	1.00	1.00				
Scaled TLI	0.98	0.97	0.98	0.98	0.97				
Robust TLI	0.94	0.89	0.96	0.95	0.92				
RMSEA	0.08	0.11	0.05	0.10	0.05				
Scaled RMSEA	0.16	0.18	0.10	0.20	0.09				
Robust RMSEA	0.15	0.16	0.09	0.14	0.09				
SRMR	0.03	0.05	0.03	0.03	0.04				
Scaled SRMR	0.03	0.05	0.03	0.03	0.04				
Reliability									
Cronbach’s α	0.85	0.83	0.82	0.89	0.95				
SB adjusted α	0.96	0.95	0.95	0.97					
$\omega_{t}$	0.93	0.89	0.88	0.94	0.97				
SB adjusted $\omega_{t}$	0.98	0.97	0.97	0.98					
$\omega_{h}$	0.85	0.83	0.78	0.87	0.93	0.02*	0.05*	0.03*	0.33*
Pearson’s *r*	0.87	0.88	0.90	0.86	0.93				
SB adjusted *r*	0.96	0.97	0.97	0.96					
ICC(2,1)	0.86	0.87	0.89	0.85	0.92				
SB adjusted ICC	0.96	0.96	0.97	0.96					

^*^Estimates reflect $\omega_{\mathrm{hs}}$ for each specific subscale that controls for variance attributable to the general factor; SB, Spearman-Brown, ACT, American Conspiracy Theory Scale, GMC, General Measure of Conspiracism, CMQ, Conspiracy Mentality Questionnaire, GCB, Generic Conspiracy Belief Scale, s., scale specific factor.

**Figure 1:**
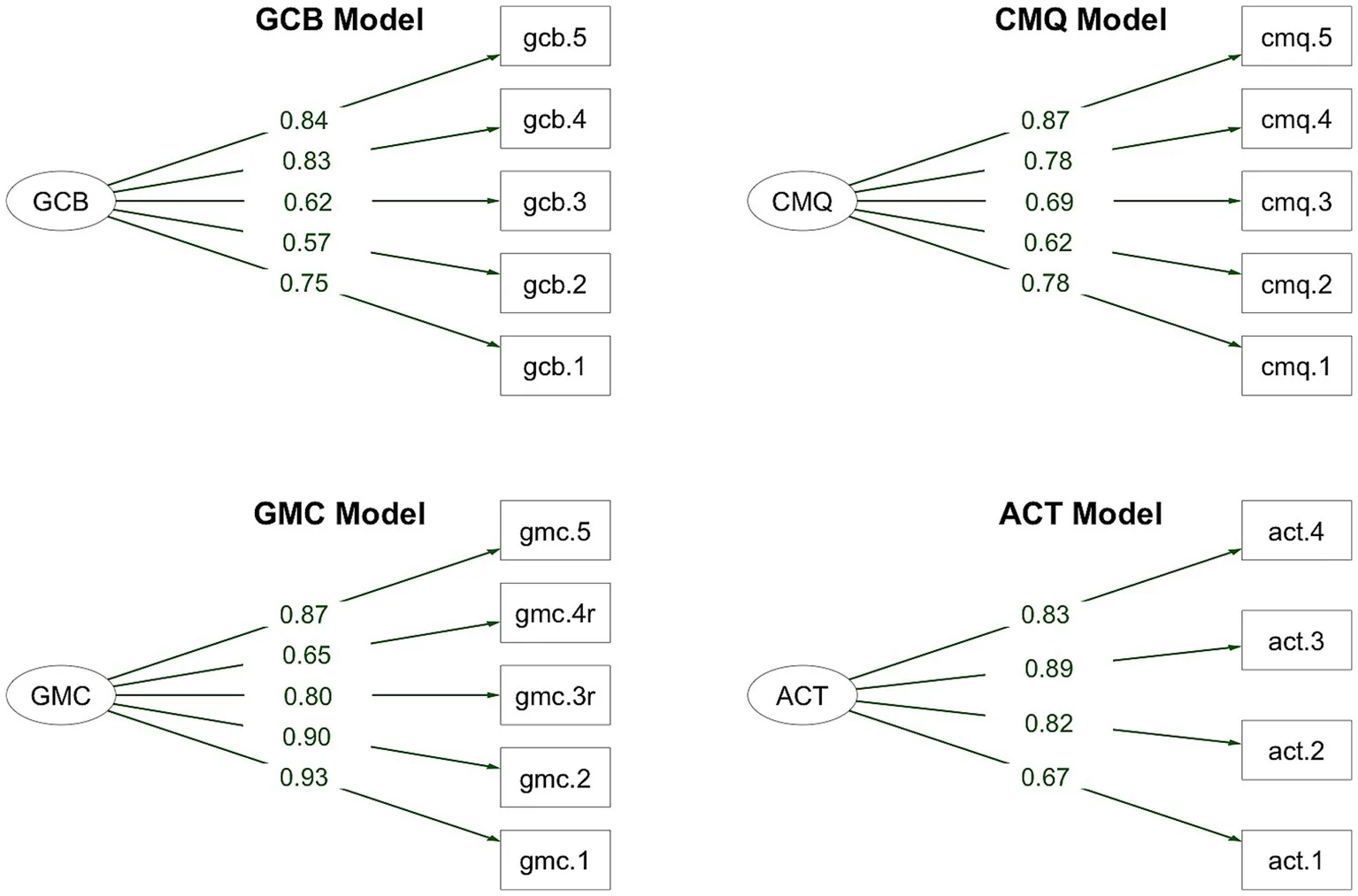
Factor loadings from four unidimensional factor models. ACT, American Conspiracy Theory Scale, GMC, General Measure of Conspiracism, CMQ, Conspiracy Mentality Questionnaire, GCB, Generic Conspiracy Belief Scale.

All fit indices for the bifactor model were either comparable or superior (particularly RMSEA) to the unidimensional factor models (see Table 4). The loadings for each item onto the general factor and the orthogonal specific factors are presented in Figure 2. Across all scales, all but two items loaded more highly on the general factor than the specific factors (CMQ.2 and GMC.4.r), but in both cases, they loaded only slightly higher (0.09 and 0.01 difference, respectively) and they both had negatively worded content. The lowest average factor loadings on the general factor were for the GMC, while average factor loadings increased incrementally for the GCB, CMC, and ACT, respectively.

**Figure 2:**
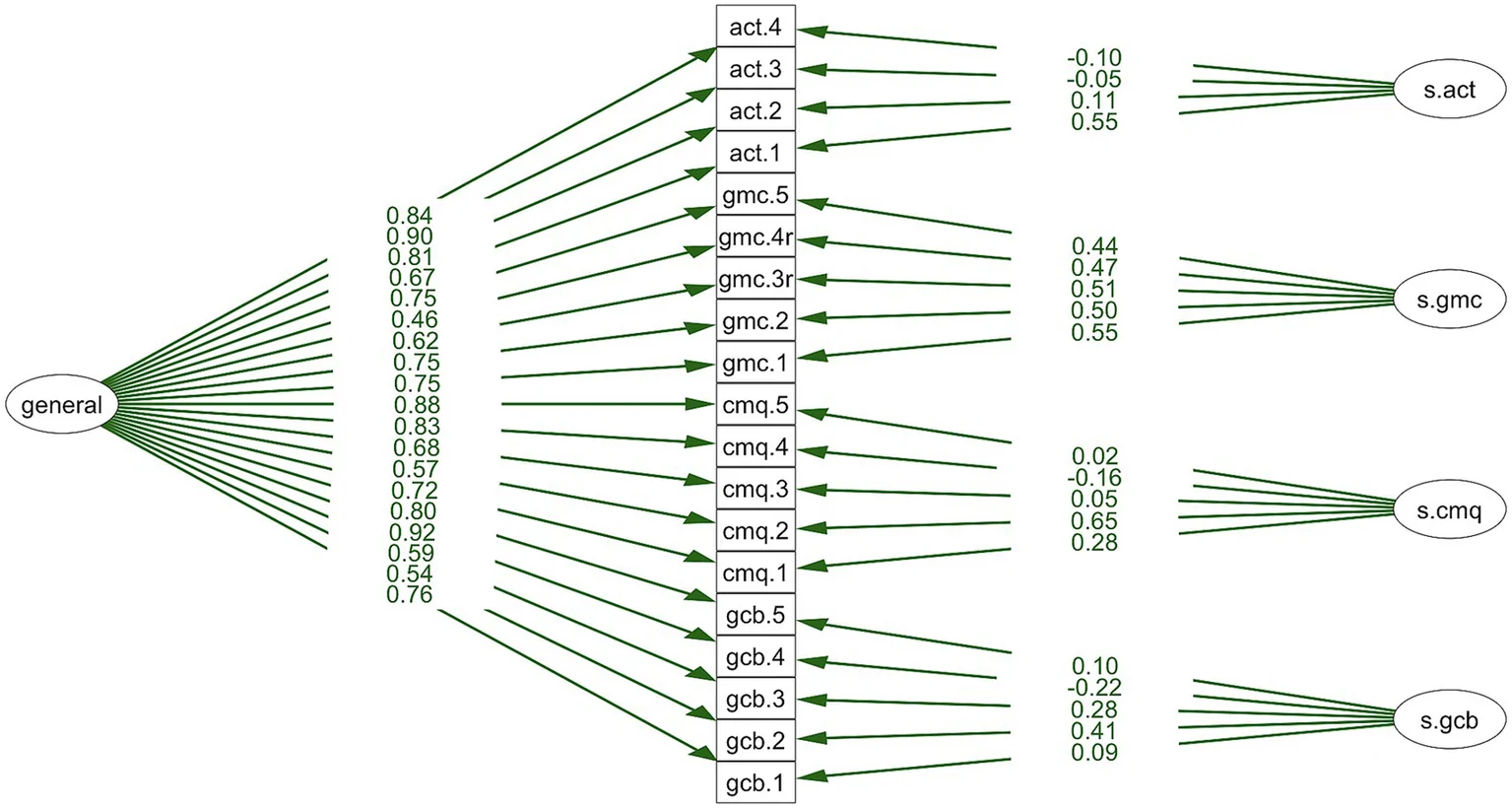
Factor loadings from bifactor model. s., specific factor, ACT, American Conspiracy Theory Scale, GMC, General Measure of Conspiracism, CMQ, Conspiracy Mentality Questionnaire, GCB, Generic Conspiracy Belief Scale.

In Figure 3, each cell above the diagonal gives the correlation between the variable named in its row and the variable names in its column. These comparisons should be read by row: the general factor’s row can be compared against the rows for each of the four scales to assess whether the general factor performs comparably well as a measure of conspiracy mentality. For example, reading across the top row, the general factor correlates at 0.76 with denialism (DEN) and at 0.78 with the BCTI. As illustrated in Figure 3, similar or superior levels of convergent and discriminant validity were observed as expected for the general factor relative to the GCB, CMQ, GMC, and the ACT when comparing correlations with DEN (strong), AGN (strong), PDS (moderate), SPQ (moderate), NFC (moderate), and IPP (weak). With respect to criterion validity, when regressing mean BCTI score on scores from the four unidimensional factor models (i.e., GCB, CMQ, GMC, and ACT) and the general factor, both $\eta_{\text{semi}-\text{partial}}^{2}$ and $\eta_{\text{partial}}^{2}$ were large for the general factor and very small or approached zero for each of the individual scales (see Table 5). This pattern indicates that the majority of the predictive value among the individual scales is shared in the general factor. In other words, the individual scales predict specific conspiracy beliefs almost entirely through what they share with one another, which raises the question of whether anything remains in their unique variances, which is our H2 below.

**Figure 3:**
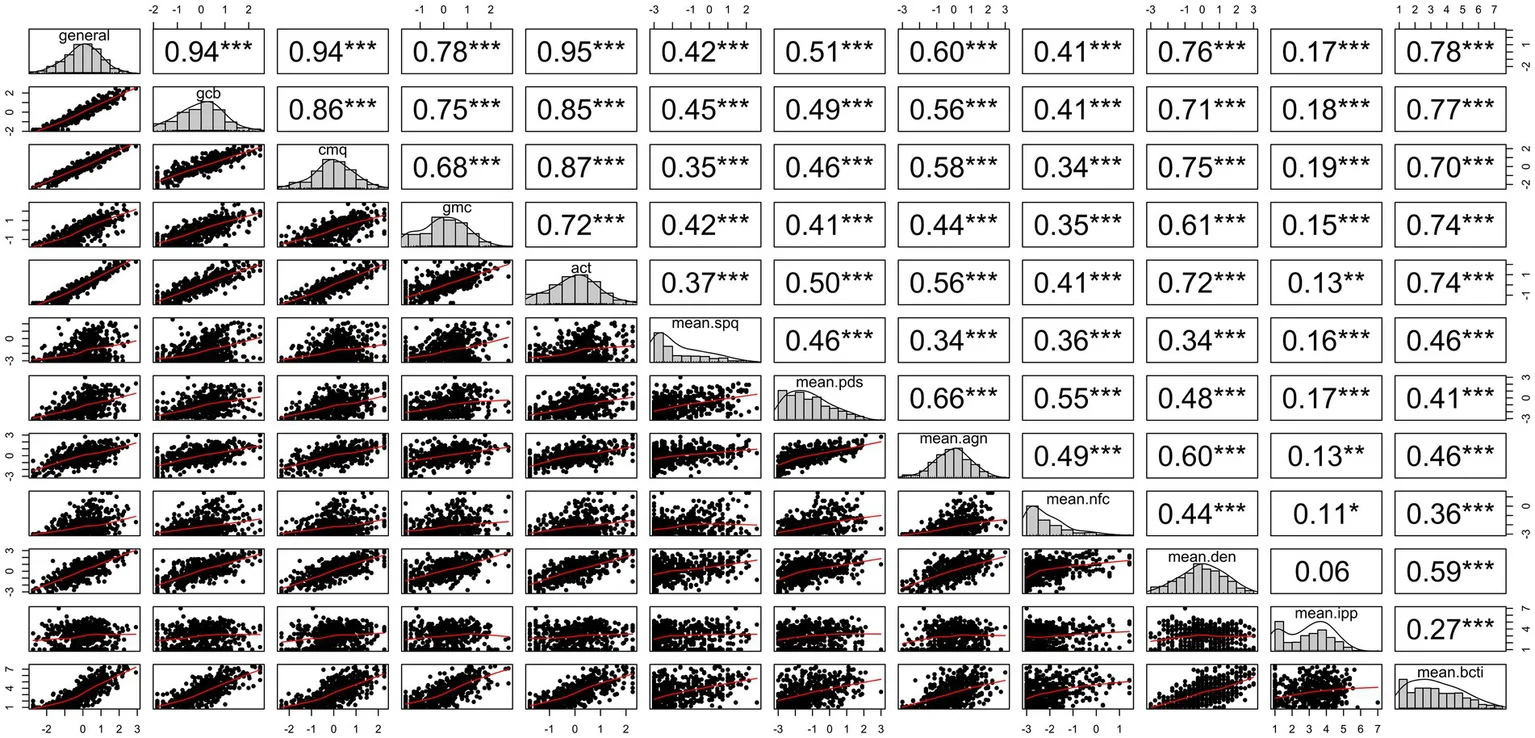
Histograms and pair-wise correlations and scatterplots with LOESS curves for scores derived from the general factor of the bi-factor model, scores from derived from the unidimensional factor models, and observed mean scores from related measures. *p < 0.05, **p < 0.01, ***p < 0.001, ACT, American Conspiracy Theory Scale, GMC, General Measure of Conspiracism, CMQ, Conspiracy Mentality Questionnaire, GCB, Generic Conspiracy Belief Scale, SPQ, Schizotypal Personality Questionnaire—Odd Beliefs Subscale, PDS, Persecution and Deservedness Scale, AGN, Agnew’s Anomie Scale, NFC, Need for Chaos Scale, DEN, Denialism Scale, IPP, Illusory Pattern Perception Scale, BCTI, Belief in Conspiracy Theories Inventory.

**Table 5:** Effect size measures from multivariable linear models regressing respondents’ mean BCTI score on predictors derived from unidimensional factor models and on predictors derived from a bifactor model.

Scores from unidimensional factor models (*R*^2^ = 0.67)			Scores from bifactor model (*R*^2^ = 0.66)		
Predictors	$\eta_{\text{semi}-\text{partial}}^{2}$	$\eta_{\text{partial}}^{2}$	Predictors	$\eta_{\text{semi}-\text{partial}}^{2}$	$\eta_{\text{partial}}^{2}$
			General factor	0.60	0.64
GCB	0.03	0.07	s.GCB	0.02	0.06
CMQ	<0.01	<0.01	s.CMQ	<0.01	0.01
GMC	0.05	0.12	s.GMC	0.03	0.07
ACT	0.01	0.03	s.ACT	<0.01	<0.01

ACT, American conspiracy theory scale; GMC, general measure of conspiracism; CMQ, conspiracy mentality questionnaire; GCB, Generic Conspiracy Belief Scale; s., scale specific factor.

As shown in Table 4, estimates of internal consistency reliability (Cronbach’s α , $\omega_{\text{total}}$ , and $\omega_{\text{hierarchical}}$ ) were uniformly higher for scores derived from the general factor than for the original ACT, CMQ, GCB, and GMC scales. However, Spearman-Brown adjustments for the shorter scales were even higher, indicating that most of the increased reliability of the general factor came from having a larger number of items. With respect to test–retest reliability, both Pearson’s r and ICC estimates were higher for scores from the general factor than for any of the original scales, but again Spearman-Brown adjustments indicate this increased reliability is likely entirely due to increased test-length.

*H2*: The residual variance in each scale-specific factor does not systematically predict individual conspiracy beliefs beyond the common factor.

Although loadings on the specific factors of the bi-factor model were generally weak and inconsistent for the ACT, CMQ, and GCB, loadings on the GMC-specific factor were consistently moderate (Table 4), suggesting that the GMC maintains a robust orthogonal residual dimension. Likewise, internal consistency reliability for the scale-specific subfactors from the bifactor model approached zero for the ACT, CMQ, and GCB—indicating negligible systematic variance beyond the general factor, but for the GMC, we again saw evidence of substantial reliable systematic variance. Taken together, this evidence suggests the GMC is measuring conspiracy mentality in an importantly different way from the other scales.

Figure 4 uses the same layout as Figure 3 to visualize correlations, but it replaces the four scale scores with their *scale-specific* residuals (i.e., s.ACT, s.CMQ, s.GCB, and s.GMC). If the second hypothesis holds for each scale, these residual rows should be near zero throughout, since each residual represents what remains of each scale once the common factor is removed. In short, any residual cell that departs from zero indicates content the common factor does not account for. For example, reading down the final column shows that, controlling for the effects of the common factor, the *residuals* of the ACT and the CMQ are essentially unrelated to the BCTI (−0.09 and −0.08, respectively) while the residuals of the GCB-5 and of the GMC remain positively related to the BCTI (0.14 and 0.23, respectively). In line with expectations, with a few exceptions, the convergent validity correlations between scores from the related measures and the scale-specific factors were generally negligible for the PDS (Persecution and Deservedness), NFC (Need for Chaos), and IPP (Illusory Pattern Perception). However, the s.GCB, s.CMQ, and s.ACT scale-specific residuals all retained weakly positive correlations with the AGN, representing social disconnectedness. Only the s.GMC retained a non-significant correlation. Both the s.CMQ and the s.ACT maintained weakly *negative* significant correlations with the SPQ, representing fewer odd beliefs or less magical thinking, while the s.GCB and the s.GMC maintained weakly positive significant correlations indicating more odd beliefs or magical thinking. The s.CMQ also maintained a weak but positive statistically significant correlation with the DEN, indicating increased suspicion of institutional information. Notably, both the s.GCB and the s.GMC continued to be predictive of the BCTI (mean number of conspiracy beliefs endorsed), suggesting additional predictive content is retained in these two scales over and above the general factor. In other words, the residuals of the ACT and the CMQ are aligned with our H2 expectation: once the common factor is removed, little systematic content remains that predicts endorsement of specific conspiracy theories. The residuals of the GCB-5 and the GMC do not, however, align with our H2 expectation, retaining associations with magical or supernatural thinking and with the BCTI over and above the common factor.

**Figure 4:**
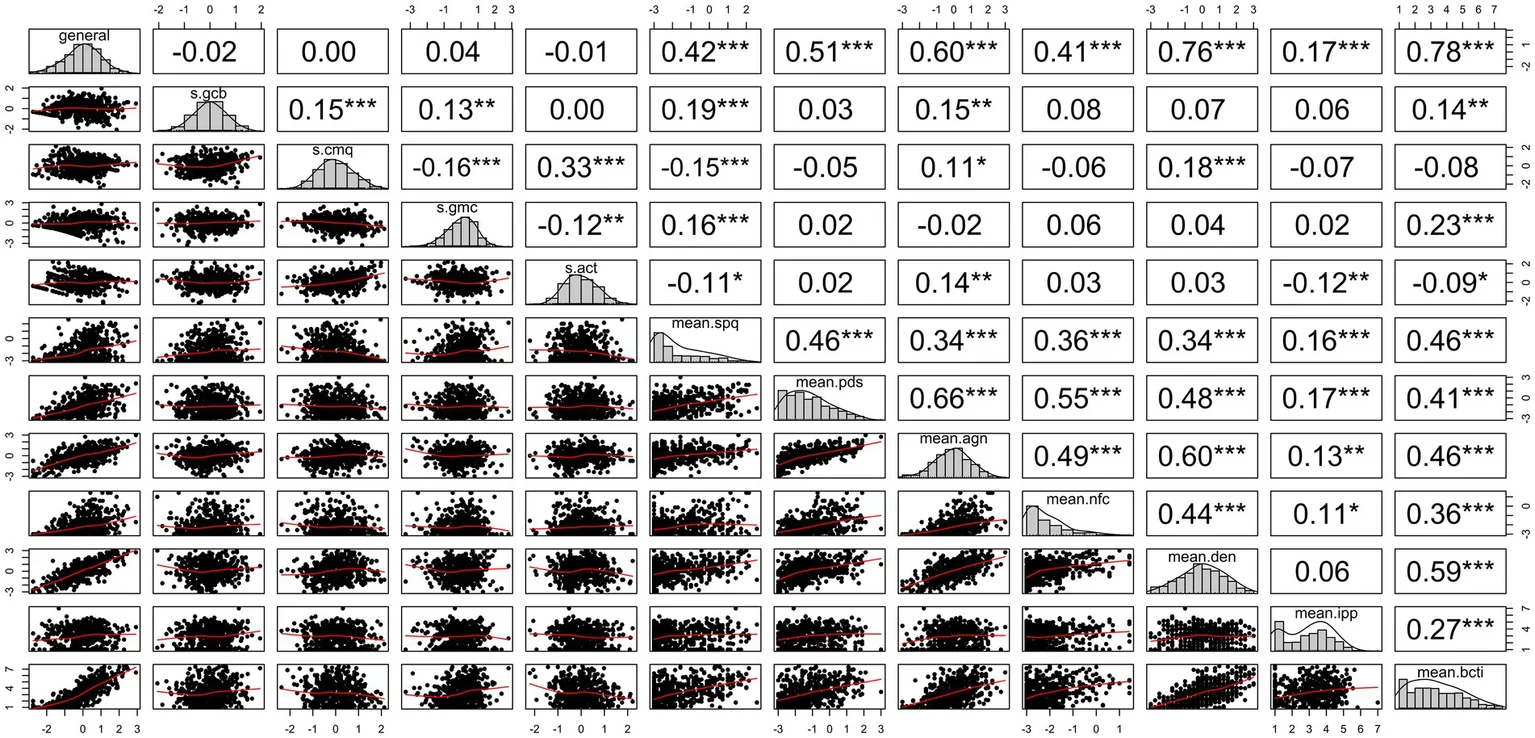
Histograms and pair-wise correlations and scatterplots with LOESS curves for scores derived from the general and specific factors of the bi-factor model and observed mean scores from related measures. *p < 0.05, **p < 0.01, ***p < 0.001, general, general factor, s., specific factor, ACT, American Conspiracy Theory Scale, GMC, General Measure of Conspiracism, CMQ, Conspiracy Mentality Questionnaire, GCB, Generic Conspiracist Belief Scale, SPQ, Schizotypal Personality Questionnaire—Odd Beliefs Subscale, PDS, Persecution and Deservedness Scale, AGN, Agnew’s Anomie Scale, NFC, Need for Chaos Scale, DEN, Denialism Scale, IPP, Illusory Pattern Perception Scale, BCTI, Belief in Conspiracy Theories Inventory.

With respect to the qualitative distinctiveness of the general and scale-specific subfactors, all items of the BCTI were more highly correlated with the general factor than with any of the scale-specific subfactors (see Figure 5). Nevertheless, small to moderate correlations persisted between the scale-specific subfactors and distinct constellations of items of the BCTI that point toward important qualitative differences. For example, the scale-specific s.GCB subfactor remained moderately correlated with 2 paranormal items of the BCTI even after controlling for the general factor and the other scale-specific subfactors (i.e., items 7 and 10). Likewise, the scale-specific s.GMC subfactor remained correlated with four partisan conspiracy theories on the BCTI after controlling for the general factor and the other scale-specific subfactors: anti-vax (Item 19), SARS as a biological weapon (Item 2), global warming (Item 20), and Obama “birther” (Item 17), all of which were salient conspiracies in recent U.S. politics during the time the data were collected in 2023 and significantly associated with Republican party identity (*r* = 0.39, 0.31, 0.50, 0.50, respectively). Given this unique partisan content among GMC items, we also carried out follow-up tests on the correlation between scores from the specific GMC factor (s.GMC) and party identity and found the s.GMC was much more positively and significantly correlated with Republican party identity (*r* = 0.28) than were the s.ACT (*r* = −0.06), s.GCB (*r* = −0.11), and s.CMQ (*r* = −0.01), all of which were either negative or statistically indistinguishable from zero.

**Figure 5:**
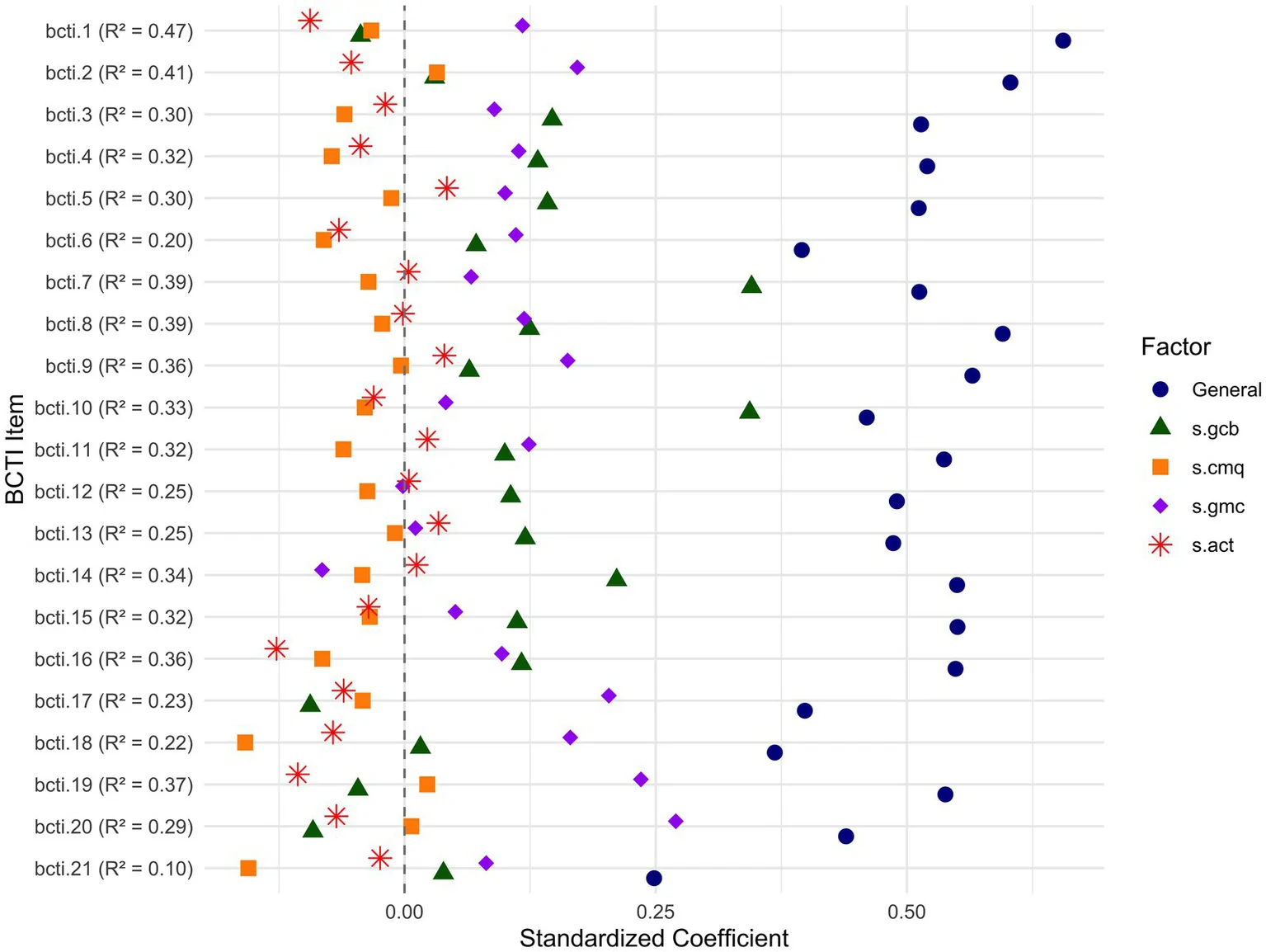
Standardized coefficients from regression models predicting individual BCTI items with scores from the general and scale-specific factors of the s.GCB, s.CMQ, s.GMC, and s.ACT.

To verify that these residual associations were not an artifact of using observed factor scores, we re-estimated the relationships between the scale-specific factors and the extraterrestrial BCTI items within the bifactor model itself. Specifically, the general and scale-specific factors were modeled as latent predictors of the observed conspiracy beliefs. The GCB-5 specific factor uniquely predicted both extraterrestrial items above and beyond the general factor (βs = 0.52 and 0.53), with effects comparable in magnitude to those of the general factor itself. In contrast, the ACT specific factor showed no meaningful association with either item (βs = 0.03 and −0.03), whereas the GMC specific factor uniquely predicted a partisan conspiracy cluster of the BCTI. Because these analyses use latent factors rather than observed scores, the findings cannot be attributed to artifacts of score derivation. If anything, the use of observed scores represents a more conservative test of our hypothesis, as measurement error in the scores would be expected to attenuate, rather than inflate, these associations.

*H3*: Each scale’s scores should be monotonically related to the common factor and should order respondents similarly across its range.

With respect to interchangeability of scores, Figure 6 plots each scale’s observed scores against observed factor scores for the general common factor. Because the general factor serves as the reference, these curves show how each scale locates respondents *relative* to the common factor. With this reference, the ACT, CMQ, and the GCB-5 assign respondents scores that correspond closely to their standing on the common factor across its range. The GMC, however, reports higher scores of conspiracy mentality by nearly 1.5 SD for those low in the trait and lower scores of conspiracy mentality by approximately 1.25 SD for those at the high end of the trait. Therefore, the GMC assigns systematically different scores to respondents at both ends of the trait relative to the general factor and all the other scales. The GMC is not strictly interchangeable with the other scales.

**Figure 6:**
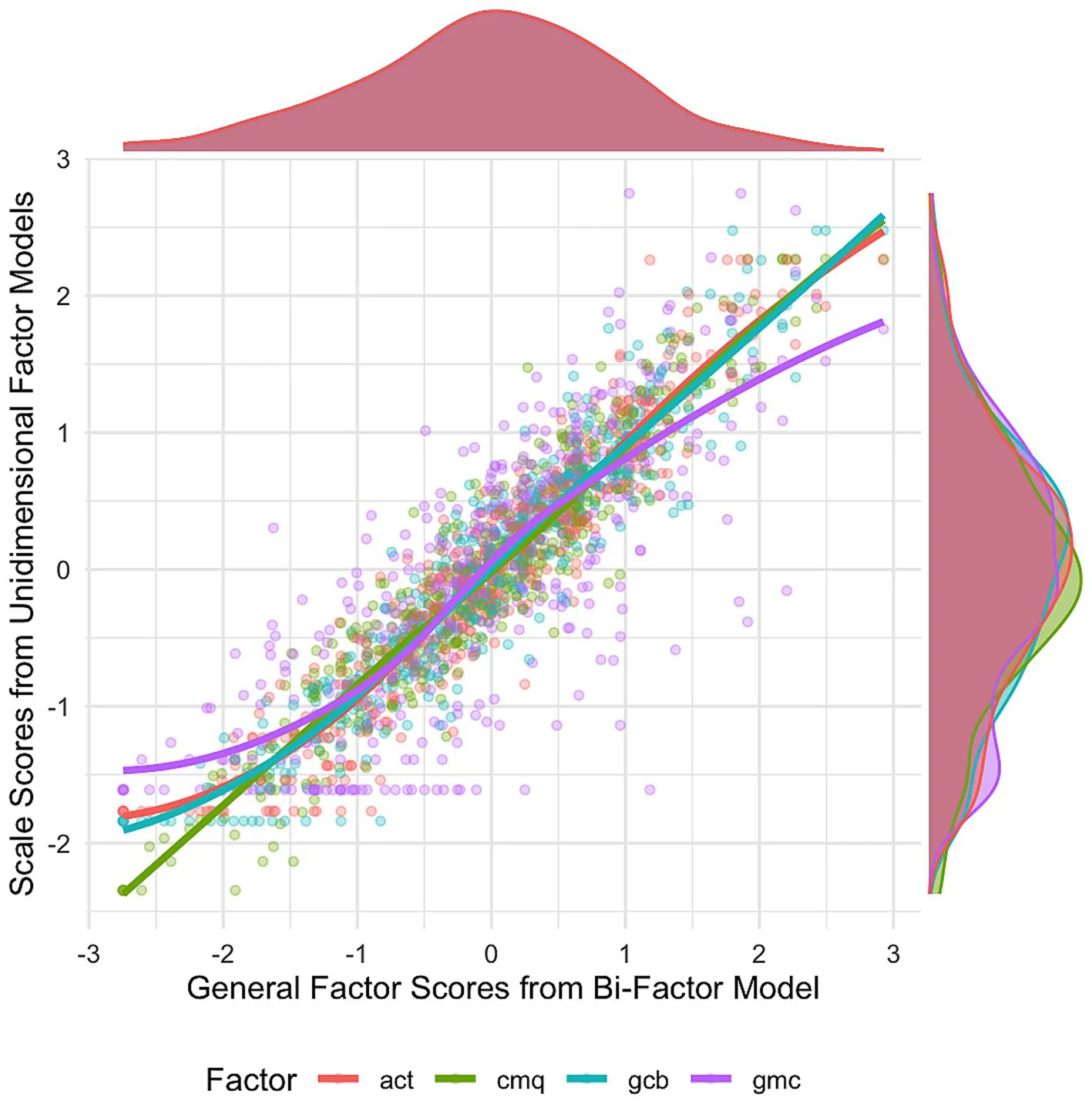
Scatterplot with LOESS curves and marginal densities of estimated general factor scores and factor scores for the ACT, CMQ, GCB, and the GMC.

## Discussion

The central question motivating this study was whether the major generic measures of conspiracy mentality are interchangeable with respect to measuring the same common latent trait, or whether their apparent similarities mask substantive differences. There are three conditions that we argued must be met before researchers should treat the ACT, CMQ, GCB-5, and the GMC as interchangeable measures of conspiracy mentality. Following the prevailing assumption of the field that conspiracy mentality is a latent, reflective trait (Imhoff et al., 2022), the first requirement for interchangeability is that each scale reflects the same underlying trait. A second requirement is that each of these scales reflect only this common trait, i.e., that it does not contain additional content that could systematically affect item endorsement (Imhoff et al., 2022, p. 3). Finally, the third requirement for interchangeability is that each scale generates similar scores representing individual differences on the underlying common conspiracy mentality factor. In other words, we argue that one cannot recommend using these scales interchangeably as measures of the latent conspiracy mentality trait unless these three conditions are met.

With respect to Hypothesis 1, we found that each of the scales studied here are reflections of an underlying common trait, in line with theoretical expectations for the conspiracy mentality construct (Imhoff et al., 2022): the common factor was best captured in items that explicitly invoked a “secret” joint collective action. Indeed, the four short-form scales reflect a general factor that accounted for most of the variance across all four instruments, and the general factor itself is related to the established correlates of conspiracy thinking (Kay and Slovic, 2026). This should be reassuring for many researchers in the field, as there have been many questions and concerns raised as to whether conspiracy mentality is a unidimensional construct (Sutton and Douglas, 2023; Uscinski and Enders, 2023; Nera, 2024a,b; Sutton et al., 2024; Trella et al., 2024). Of course, these findings cannot adjudicate whether conspiracy mentality should be theoretically a unidimensional or multidimensional construct. Instead, regardless of these emerging theoretical concerns, our findings do suggest that the popular empirical measures of the generic conspiracy mentality trait are measuring the same unidimensional construct. This is the important first step for establishing interchangeability of the scales, since we now have good reason to understand each scale to be measuring the same latent trait. It is not, however, sufficient. A scale can reflect the common trait and still fail to be interchangeable with other scales either by carrying systematic content beyond the shared trait, or by differentially reporting how much of that trait a respondent has, which are our second and third hypotheses, respectively.

Given the reflective model assumption, after we partitioned the shared and unique variance from each of the four generic scales, we hypothesized that the unique variance should be unrelated to specific conspiracy belief endorsement (H2). That is, given the unique ways that the generic scales were developed, while each scale may have some unique residuals, these unique differences should be unrelated to specific conspiracy theory endorsement. Where we had uniform success with H1, H2 held for only two of the four scales. After controlling for the common factor, the residuals of the ACT and the CMQ were unrelated to the BCTI. However, the residuals for the GCB-5 and the GMC were related to the BCTI. While their contribution to predicting overall BCTI scores was small, each of their effects were localized to a particular subset of the items. These associations were not a product of observed scores, either. When the general and scale-specific factors were entered as latent predictors within the bifactor model, for example, the GCB-5’s specific factor predicted the two extraterrestrial items in the BCTI as strongly as the general factor (β = 0.52 and 0.53, respectively). Following the reflective model assumption, this is evidence that the GCB-5 and the GMC are not strictly interchangeable with the ACT and the CMQ. The nature of the variance within these two scales raises a larger theoretical question, which we discuss more below.

Finally, where the second hypothesis focused on the relationship between outcomes correlated with the unique residuals of each scale and with the common factor, the third hypothesis, and condition of interchangeability, focuses on the relationship between each original scale and the common factor directly: each scale should be monotonically related to the common factor and should order respondents similarly across the general trait. Since we assume that each scale should reflect only what is held in common to the other scales, we plotted each scale’s scores against general factor scores and found that the ACT, CMQ, and the GCB-5 followed the common factor across its range. The GMC, however, produced more extreme values at both ends of the general conspiracy mentality, diverging from the common factor by roughly 1.5 standard deviations among respondents low on the general trait and by roughly 1.25 standard deviations among those on the high end of the trait. In other words, two respondents with very different general trait scores are assigned more *similar* GMC scores than their true difference warrants. As such, the GMC fails the third requirement for interchangeability.

The findings presented here suggest that the recommendation that researchers can use these scales almost interchangeably is not warranted. While the four short-form scales do measure the same latent conspiracy mentality trait, two of the scales introduce two different complications that make them not strictly interchangeable with each of the other scales. Turning to the GMC, unlike the other scales, the items retained consistently moderate loadings on its residual factor. This residual was positively associated with Republican party identity (*r* = 0.28), while the other scales had a null or negative association. While the conspiracy mentality trait may be related to conservative or Republican identity (Enders et al., 2023), our findings suggest that the GMC’s residual, after the common factor has been removed, contains an additional partisan association that none of the other scale residuals share. Moreover, the GMC residual factor continued to predict a small distinct cluster of BCTI items after controlling for the general factor that mirror largely Republican-held beliefs in 2023: beliefs about SARS as a bioweapon, the Obama “birther” conspiracy theory, anti-vaccine claims, and that global warming was a hoax (see Table 2, BCTI items 2, 17, 19, and 20) (Smallpage et al., 2017; Enders et al., 2020). That the GMC asks about the label “conspiracy theory” can be wielded by and against political outsiders (Uscinski and Parent, 2014; Uscinski et al., 2016; Uscinski and Enders, 2023), and the fact that it captures Republican beliefs (given the timing of the study) is perhaps, upon retrospect, expected. As we discuss briefly in the Limitations section below, whether this is an artifact of the American context in 2023 or a feature of the GMC itself, cannot be determined from a single cross-sectional sample. Nevertheless, the GMC contains something not found in the three other scales we analyzed.

As noted above, after controlling for the common factor, the GCB-5’s residual factor was significantly and positively associated with the SPQ, a measure of odd and magical thinking, which is an external construct different from the construct of conspiracy mentality (Sutton and Douglas, 2023). The residual factor also predicted a subset of the BCTI, after controlling for the common conspiracy mentality factor: the two extraterrestrial items about a government cover up of an alien crash in Roswell, New Mexico (item 7) and the existence of Area 51 (item 10). In short, these items are face valid indicators of the paranormal theme contained within the GCB-5. Taken together, these findings suggest that the GCB-5 is capturing a respondent’s propensity to see the world filled with secrets *and* the respondent’s belief that some of these secrets are about paranormal phenomena.

We began this paper with the observation that conspiracy theory researchers have continued to adopt the use of general measures of conspiracy mentality. Rather than relying on specific conspiracy theory endorsement, researchers use generic scales because the endorsement of a specific conspiracy theory may be “content contaminated” insofar as a respondent may endorse a conspiracy theory either because of their underlying conspiracy mentality trait or because of some other motivation. For example, prior work has demonstrated that some people may endorse a specific conspiracy theory because of its partisan content (Smallpage et al., 2017; Enders et al., 2020). In our data, the Obama “birther” conspiracy is correlated with the common conspiracy mentality trait, but it is also correlated with the GMC residual factor that correlated with Republican party identification. The same is also true for the GCB-5 but with paranormal content and the extraterrestrial items of the BCTI. Like specific conspiracy items, some of the generic scales may share a pattern consistent with content-contamination as Imhoff et al. (2022) describe it.

At best, the “content-contamination” interpretation of our findings should be understood as provisional. The same patterns may, instead, be consistent with an alternative interpretation: that is, the GCB-5 and the GMC may be capturing *more conspiracy mentality*, not something else entirely. On this view, these generic scales may be covering a wider landscape of conspiracy theories that the other scales are not (Kay and Slovic, 2026). Indeed, as discussed above, the GCB-5 and the GCB-15 were designed to capture the “breadth” of specific conspiracy theories, which include extraterrestrial coverups. That some of these scales have a stronger association with endorsing specific conspiracy theories would be a virtue and not a defect. Nevertheless, we recognize that the present design and analyses cannot settle the substantive question of whether the non-interchangeability among these scales is due to “content contamination” or not. Minimally, the evidence contained in this paper raises this as an important question for future research to investigate.

The question this paper can settle, however, is whether the four short-form scales of generic conspiracy mentality should be treated as empirically interchangeable. The scales cannot be treated as interchangeable measures of the same latent conspiracy mentality trait. This is true regardless of whether one takes the GCB-5 or the GMC to be “content-contaminated” or if one believes these two scales capture more endorsement of paranormal and partisan conspiracy beliefs, respectively. Therefore, practically, researchers should treat the selection of a generic conspiracy mentality scale as a deliberate, nontrivial research decision, and should justify that choice in light of their research question. For researchers who want to conduct studies across pooled samples that use different measures for conspiracy mentality, our findings suggest that there should be caution in assuming these are equivalent estimates across studies. Researchers collecting their own survey samples should be aware of how these scales differ from one another. More concretely, researchers who use the GCB-5 should be aware that its scores may be carrying paranormal ideation alongside conspiracy mentality, and at a minimum we recommend including a measure of paranormal or magical thinking to gauge its influence on whatever outcome of interest. For those researchers who use the GMC, we likewise recommend including a measure of political partisanship or political ideology. We also recommend more caution should be used when using the GMC as it may not only carry additional partisan content that the other scales do not, but its scores also locate respondents on the underlying common conspiracy mentality trait differently from the other scales, particularly at the extremes. The four short-form scales of conspiracy mentality we have measured here are not strictly interchangeable with one another.

## Limitation and further research

While this study has a considerable number of strengths, it does have multiple limitations. First, our estimates come from a single cross-sectional sample – a secondary analysis of Kay and Slovic’s (2026) Prolific study. While Prolific samples tend to outperform other online survey panels on attentiveness, comprehension, and the reproduction of established experimental and correlational findings (Peer et al., 2017, 2022; Douglas et al., 2023), the sample still comes from a single Western, Educated, Industrialized, Rich, and Democratic (WEIRD) country at a single point in time. In this sense, further original research capturing these measures across cultures and countries is needed to assess the reliability and generalizability of the findings. Further, the data used here are vulnerable to the same limitations previously outlined by Kay and Slovic (2026): the dataset does not include all extant general measures of the conspiracy mentality; it includes only some measures for convergent validity and relies especially on the BCTI as its measure of criterion validity.

When it comes to the limited use of measures, further research should investigate how generalizable these findings are across alternative measures of convergent and criterion validity, other specific conspiracy theories beyond those contained in the BCTI-21, and other general measures of conspiracy mentality, such as the Conspiracy Mentality Scale (Stojanov and Halberstadt, 2019). Such additional data collection efforts would also allow researchers to assess the extent to which the pattern that we found in the GMC and GCB-5 is indeed reflected in the item content, or an artifact of this cross-sectional study. For example, until further analysis by other researchers either corroborates or challenges our findings, we cannot know whether the partisan residual factor of the GMC is unique to the highly polarized American political climate in 2023 or is a feature of the GMC as such, i.e., in different contexts. This study also deployed only the short-form versions of multiple scales, so the findings in our paper require caveats until a similar analysis is replicated with full item scales. This is particularly important when it comes to assessing the generalizability of what we found in the GCB-5 to the GCB-15, which we did not have access to in this dataset. Given that the GCB-5 is a validated measure derived from the items that loaded strongest on each of the GCB-15’s five thematic aspects, we would not expect our findings using the GCB-5 to be completely missing in the GCB-15, but, of course, this is an empirical question.

The findings of this study are also limited in terms of the methods we deployed. We did not test for temporal invariance of the factor structure of the general factor nor of any of the other previously validated scales used in this study. This would be a critical step before using the items to form a new general factor for use in future research going forward. Moreover, we used a two-step method to test correlations between observed factor scores and observed scores from related measures (i.e., we derived factor scores first using the EBM method and then calculated Pearson’s *r* and *ICC* instead of estimating the correlations among the latent variables directly using SEM). There are a variety of reasons we adopted the simpler two-step approach, both theoretical (i.e., we are not aiming to validate any specific scale) and practical (multiplicative growth in the number of parameters, model complexity, and the associated estimation problems like we saw when attempting to estimate the correlated factors model). Nevertheless, when using the two-step method, correlations are systematically downward biased, Spearman (1904) suggesting that the correlations we report between the scale-specific factors and ancillary paranormal and partisan content have been *under*-estimated.

Further research will be needed to adjudicate the question that we have raised in this paper: are the residual factors we found in the GCB-5 and the GMC marks of “content-contamination” or “breadth”? This paper has established the precondition to this investigation: that there are empirical differences among the generic scales that do not warrant treating them as interchangeable. A future study could include outcomes where paranormal ideation would be positively associated but conspiracy mentality not, and vice versa, and see the extent to which the GCB and the other scales behave differently across them. Perhaps more directly, an experimental study could vary the paranormal content of a conspiracy theory, which would reveal if there are any differences among the generic belief scales. Designing this study, however, also raises the question of how one defines or distinguishes a conspiracy theory from other kinds of related beliefs (Sutton and Douglas, 2023).

On a more abstract level, this study has assumed that conspiracy thinking is its own unique trait—"conspiracy mentality.” Recent research, however, has shown that there is significant overlap among “unwarranted epistemic beliefs” like conspiracy thinking, paranormal beliefs, and beliefs in pseudoscience (Lobato et al., 2014; Smallpage et al., 2023). This study focused only on partitioning the general and unique variance among measures of conspiracy mentality in isolation from these other overlapping beliefs, and further research is needed to examine whether this theoretical and empirical approach is warranted. Preliminary evidence from a 2023 study suggests there may be a single underlying “romantic” (i.e., anti-Enlightenment) orientation towards evidence and ethics of belief that drives this cluster of epistemically unwarranted belief sets and their effects on authoritarian political commitments (Smallpage et al., 2023). Regardless, this study has shown that, if conspiracy mentality is a theoretically coherent construct, there is strong empirical evidence that some of the general measures studied here reflect that general construct, though they are not practically interchangeable with each other.

Further research should explore the underlying latent common factor of conspiracy mentality more than we have been able to do in this paper. For example, the common factor was best reflected by a few items from three of the four generic scales. Using the bifactor evidence from this paper, future research could develop a more robust and conceptually discrete measure of the general factor of conspiracy mentality. Clarifying the contours of the general latent psychological trait of conspiracy mentality is therefore the most important project for those of us examining the causes, correlates, and consequences of conspiracy theory beliefs.

## Data Availability

Publicly available datasets were analyzed in this study. This data can be found at: https://osf.io/kzr27/overview.

## Footnotes

1
https://osf.io/uzrgk/overview
